# Signal Transduction of Transient Receptor Potential TRPM8 Channels: Role of PIP5K, Gq-Proteins, and c-Jun

**DOI:** 10.3390/molecules29112602

**Published:** 2024-06-01

**Authors:** Gerald Thiel, Oliver G. Rössler

**Affiliations:** Department of Medical Biochemistry and Molecular Biology, Medical Faculty, Saarland University, 66421 Homburg, Germany; oliver.roessler@uks.eu

**Keywords:** c-Jun, G-protein, Gαq-coupled receptor, gallein, ISA-2011B, phosphatidylinositol 4-phosphate 5 kinase, RGS2, TRPM3, TRPM8

## Abstract

Transient receptor potential melastatin-8 (TRPM8) is a cation channel that is activated by cold and “cooling agents” such as menthol and icilin, which induce a cold sensation. The stimulation of TRPM8 activates an intracellular signaling cascade that ultimately leads to a change in the gene expression pattern of the cells. Here, we investigate the TRPM8-induced signaling pathway that links TRPM8 channel activation to gene transcription. Using a pharmacological approach, we show that the inhibition of phosphatidylinositol 4-phosphate 5 kinase α (PIP5K), an enzyme essential for the biosynthesis of phosphatidylinositol 4,5-bisphosphate, attenuates TRPM8-induced gene transcription. Analyzing the link between TRPM8 and Gq proteins, we show that the pharmacological inhibition of the βγ subunits impairs TRPM8 signaling. In addition, genetic studies show that TRPM8 requires an activated Gα subunit for signaling. In the nucleus, the TRPM8-induced signaling cascade triggers the activation of the transcription factor AP-1, a complex consisting of a dimer of basic region leucine zipper (bZIP) transcription factors. Here, we identify the bZIP protein c-Jun as an essential component of AP-1 within the TRPM8-induced signaling cascade. In summary, with PIP5K, Gq subunits, and c-Jun, we identified key molecules in TRPM8-induced signaling from the plasma membrane to the nucleus.

## 1. Introduction

TRPM8 (transient receptor potential melastatin-8) was identified as a menthol receptor using an expression cloning approach [1]. Menthol, a p-menthane-3-ol derived from the oil of peppermint, is known to induce a cold sensation. Analysis of the primary structure revealed that the receptor belongs to the TRP family of cation channels. The TRPM8 cDNA was simultaneously identified using a genomic DNA database search and PCR from a dorsal root ganglion cDNA library [2]. The TRPM8 channel is activated by cold temperature and cooling substances such as menthol, eucalyptol, and the synthetic “super-cooling agonist” icilin [3]. TRPM8, like other TRP channels, is a polymodal sensor that integrates temperature and chemical sensations and plays an essential role in thermosensation, as shown by an analysis of TRPM8-deficient mice [4,5,6]. TRPM8 is found in sensory neurons, where it functions as a cold nociceptor which mediates nocifensive responses to noxious cold [5,6]. In addition, TRPM8 channels are involved in cold hypersensitivity triggered by nerve injury and inflammation. TRPM8 channels are also thought to be involved in the development of migraine, the development of tumors, and other diseases [7,8,9]. An anti-inflammatory role has also been proposed for TRPM8 [10,11].

The stimulation of TRPM8 channels triggers an intracellular signaling pathway that leads to a change in the gene expression pattern of the cells. We are interested in identifying the signaling molecules that are essential for the link between TRPM8 stimulation and gene transcription. Previous studies have shown that an influx of Ca^2+^ ions is essential for the continuation of the signaling cascade after TRPM8 stimulation [12]. The extracellular signal-regulated protein kinase ERK1/2 was identified as an intracellular signal transducer [13]. In addition, calmodulin, calcineurin, and phospholipase C (PLC) β have been identified as important molecules that enable signal transduction from the plasma membrane to the nucleus after TRPM8 channel stimulation [14,15]. However, the TRPM8-induced signaling cascade is far from being described in detail.

In this study, we focused our attention on the roles of phosphatidylinositol 4,5-bisphosphate and trimeric G-protein subunits within the TRPM8-induced signaling cascade. Several reports have described the regulation of TRPM8 channels by phosphatidylinositol 4,5-bisphosphate [16,17,18,19,20,21], based on genetic tools that induced the dephosphorylation of phosphatidylinositol 4,5-bisphosphate. We explored whether we could attenuate the signaling pathway of TRPM8 by interfering with the biosynthesis of phosphatidylinositol 4,5-bisphosphate.

It has been suggested that G-proteins modulate TRPM8 signaling, although this issue remains controversial. The stimulation of Gαq-coupled receptors activates phospholipase C, which catalyzes the hydrolysis of phosphatidylinositol 4,5-bisphosphate, thereby reducing phosphatidylinositol 4,5-bisphosphate levels. However, the stimulation of Gαq-coupled receptors also leads to a rise in cytosolic Ca^2+^ and the activation of protein kinase C and other protein kinases that can influence channel activity. Direct binding of the Gαq subunit to TRPM8 has been shown [22], but it is controversial whether the activated Gαq stimulates or inhibits TRPM8 [22,23] and whether phospholipase C is involved or not. In this study, we used pharmacological and genetic strategies to elucidate the roles of the α and βγ subunits of Gq-coupled receptors in TRPM8-mediated signaling.

Recently, we showed that the stimulation of TRPM8 channels activates the transcription factor AP-1 [13,24]. AP-1 is composed of two basic region leucine zipper (bZIP) transcription factors of the Jun, Fos, and ATF families of transcription factors. We asked which bZIP proteins are involved in the genetic changes in the nucleus after the stimulation of TRPM8 channels.

## 2. Results

### 2.1. Biosynthesis of Phosphatidylinositol 4,5-Bisphosphate

**Figure 1:**
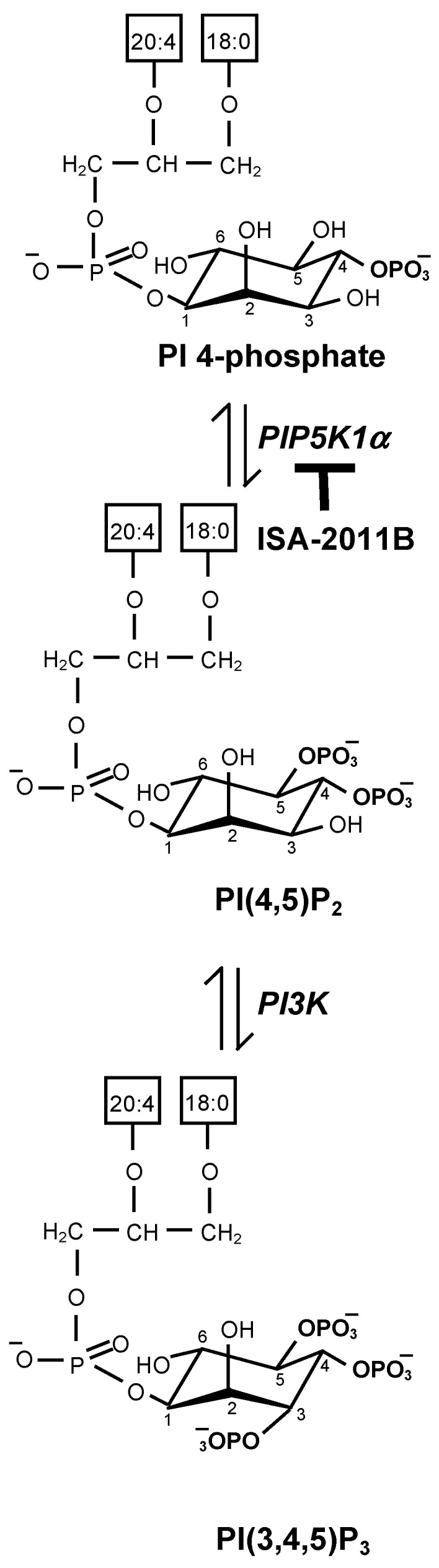
Biosynthesis, metabolism and hydrolysis of phosphatidylinositol 4,5-bisphosphate. Phosphoinositides are phosphorylated metabolites of phosphatidylinositol. Catalyzed by PIP5K, phosphatidylinositol 4,5-bisphosphate (PI(4,5)P_2_) is synthesisized from phosphatidylinositol 4-phosphate (PI 4-phosphate). Phosphatidylinositol 4,5-bisphosphate can be phosphorylated by phosphatidylinositol 3-kinases to generate phosphatidylinositol 3,4,5-trisphosphate (PI(3,4,5)P_3_), a lipid mediator essential to activate the serin/threonine protein kinase AKT. Phosphatidylinositol 4,5-bisphosphate can also be metabolized by phospholipase C (PLC), which generates IP_3_ and diacylgycerol.

Phosphatidylinositol 4,5-bisphosphate, an important lipid involved in the control of numerous signaling pathways, is mainly synthesized from phosphatidylinositol 4-phosphate [25], a reaction catalyzed by the enzyme PIP5K, which performs the transfer of a phosphate group to the 5′-position of the inositol ring (Figure 1). Phospholipase C enzymes catalyze the hydrolysis of phosphatidylinositol 4,5-bisphosphate to generate the second messengers IP_3_ and diacylglycerol. Phosphatidylinositol 4,5-bisphosphate further functions as a substrate for phosphatidylinositol 3-kinases, which catalyze the transfer of a phosphate group to the 3′-position of the inositol ring, thus generating phosphatidylinositol 3,4,5-trisphosphate (Figure 1), a metabolite which is essential for the activation of the phosphoinositide-dependent protein kinase AKT.

### 2.2. Pharmacological Inhibition of Phosphatidylinositol 4,5-Bisphosphate Biosynthesis Interferes with Signaling via the TRPM8 Channel

**Figure 2:**
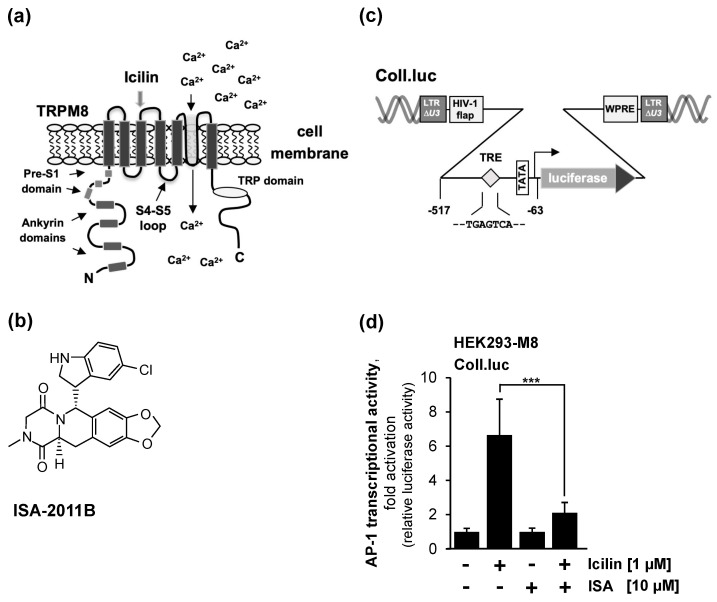
Pharmacological inhibition of PIP5Kα attenuates TRPM8 intracellular signaling. (**a**) Modular structure of TRPM8. The interaction sites of phosphatidylinositol 4,5-bisphosphate with the channel are shown (preS1 segment, S4-S5 linker, TRP domain). (**b**) Chemical structure of the PIP5Kα inhibitor ISA-2011B. (**c**) Luciferase reporter gene under control of the collagenase promoter (Coll.luc), which serves as a sensor for measuring AP-1 activity. Shown is the chromatin-embedded provirus, which also contains the Woodchuck hepatitis virus posttranscriptional regulatory element (WPRE) and the HIV flap element. The U3 region of the 5′ LTR has been deleted. (**d**) HEK293-M8 cells were infected with a recombinant lentivirus containing the Coll.luc reporter gene. Cells were serum-starved for 24 h, preincubated for 3 h with ISA-2011B (10 μM), and then stimulated with icilin (1 μM) in serum-reduced medium in the presence of the PIP5Kα inhibitor. Cell extracts were prepared, and luciferase activities and protein concentrations were determined. The luciferase activity was normalized to the protein concentration. Data shown are means +/− SD of three experiments performed in quadruplicate (*** *p* < 0.001).

Figure 2a shows the modular structure of TRPM8 channels, revealing the typical architecture of TRP channels with six transmembrane domains and both N- and C-termini in the cytoplasm. The figure also shows the proposed interaction sites of phosphatidylinositol 4,5-bisphosphate with the TRPM8 ion channel [26].

A variety of approaches have been used to manipulate the levels of phosphatidylinositol 4,5-bisphosphate in the plasma membrane, including the Gαq-coupled receptor-mediated activation of phospholipase C, the expression of a voltage-dependent lipid phosphatase, or the administration of various compounds designed to reduce phosphatidylinositol 4,5-bisphosphate levels. In this study, we used a pharmacological approach to inhibit the biosynthesis of phosphatidylinositol 4,5-bisphosphate by incubating the cells with the PIP5Kα inhibitor ISA-2011B (Figure 2b). This compound has been shown to significantly inhibit PIP5K activity and block the subsequent activation of AKT [27,28].

The stimulation of various TRP channels (TRPC6, TRPM3, TRPM8, and TRPV1) leads to the activation of the transcription factor AP-1 [24,29,30,31]. In addition, AP-1 is activated by the stimulation of voltage-gated Ca_v_1.2 Ca^2+^-channels and Gαq-coupled receptors [32,33]. We investigated the signaling pathway from the plasma membrane to the nucleus and used the activation of AP-1 as a measure for the nuclear response to TRPM8 stimulation. Frequently, calcium imaging techniques and/or patch-clamp electrophysiology are used as an indicator for TRPM8 activation. Our chosen approach has the advantage of tracing a TRPM8-induced intracellular signaling cascade from the plasma membrane to the nucleus. A collagenase promoter/luciferase reporter gene, shown in Figure 2c, was used as a sensor to detect changes in AP-1 activity [34]. This transcription unit contains an AP-1 binding site, also known as an 12-*O*-tetradecanoylphorbol-13-acetate (TPA)-responsive element (TRE), in the proximal promoter region. The reporter gene was integrated into the chromatin of the cells using lentiviral gene transfer. This strategy ensured that the reporter gene was embedded into a nucleosomal structure. HEK293-M8 cells were infected with a lentivirus containing the Coll.luc reporter gene. The cells were preincubated with the compound ISA-2011B for 3 h and then stimulated for 24 h with icilin in the presence of the PIP5K inhibitor. Figure 2d shows that the stimulation of the HEK293-M8 cells with icilin increased the AP-1 activity by 6.7-fold, while the administration of ISA-2011B strongly inhibited intracellular TRPM8 signaling, resulting in only a 2.1-fold increase in AP-1 activity. Thus, AP-1 activity was reduced by 81% in the presence of ISA-2011B.

### 2.3. Pharmacological Inhibition of Phosphatidylinositol 4,5-Bisphosphate Biosynthesis Interferes with Signaling via the TRPM3 Channel

**Figure 3:**
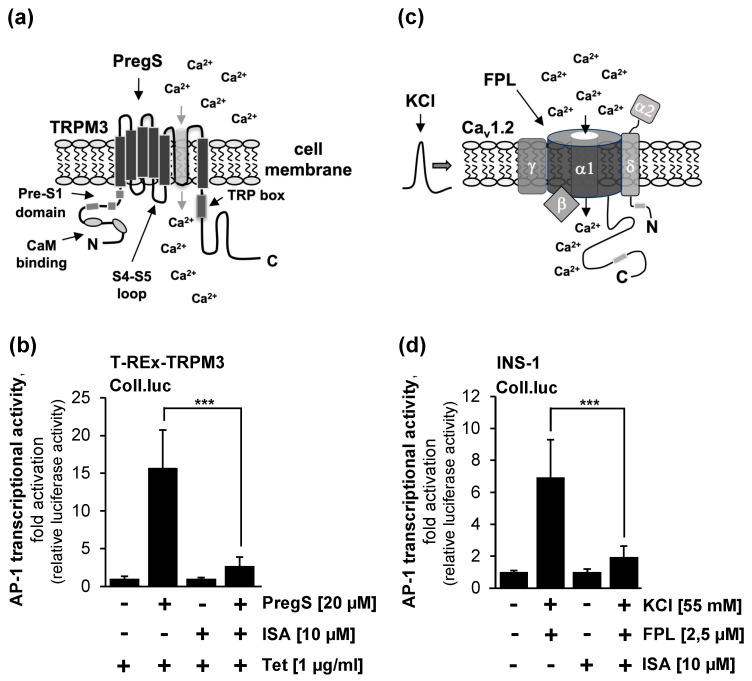
Pharmacological inhibition of PIP5Kα attenuates intracellular signaling following stimulation of TRPM3 and Ca_v_1.2 Ca^2+^ channels. (**a**) Modular structure of TRPM3 showing the interaction sites of phosphatidylinositol 4,5-bisphosphate with the channel (preS1 segment, S4-S5 linker, and TRP domain). (**b**) T-REx-TRPM3 cells containing a Coll.luc reporter gene integrated into the chromatin were serum-starved for 24 h in the presence of tetracycline (1 μg/mL) to induce TRPM3 expression. The serum-starved cells were preincubated with ISA-2011B (10 μM) for 3 h, and then stimulated with pregnenolone sulfate (20 μM) for 24 h in the presence of the inhibitor. Cells were harvested and analyzed as described in the legend to Figure 2 (n = 4; *** *p* < 0.001). (**c**) Modular structure of Ca_v_1.2 voltage-gated Ca^2+^ channels, consisting of the α1 subunit, which forms the pore, and the auxiliary subunits α2δ, β, and γ. (**d**) INS-1 832/13 insulinoma cells were infected with a recombinant lentivirus containing the Coll.luc reporter gene. Cells were serum-starved in medium containing 0.5% serum and 2 mM glucose for 24 h. Cells were preincubated in the same medium with ISA-2011B (10 μM) for three hours. Stimulation of the cells was performed with KCl (25 mM) and the voltage-gated Ca^2+^ channel activator FPL64176 (2.5 μM) in the presence of the inhibitor for 24 h. Cells were harvested and analyzed as described in the legend to Figure 2 (n = 3; *** *p* < 0.001).

As a control, we examined the effect of ISA-2011B application on the activation of another TRPM cation channel, TRPM3. TRPM3, similar to TRPM8, is a polymodal channel that can be activated by heat and chemicals such as the steroid pregnenolone sulfate [35,36]. The activation of TRPM3 has been linked to heat and pain sensations, gene transcription, vascular smooth muscle contraction, insulin secretion, and tumorigenesis [36]. A recent analysis of gain-of-function mutations of TRPM3 revealed a role of this ion channel in chronic fatigue syndrome/myalgic encephalomyelitis and in the development of neuronal disorders [37,38]. Using cell-free inside-out patches, TRPM3 activity was shown to increase in response to phosphoinositides, and was reduced after the expression of a voltage-sensing phosphatase [21,39]. Figure 3a shows the modular structure of TRPM3, including its proposed interaction sites with phosphatidylinositol 4,5-bisphosphate. Figure 3b shows that the stimulation of T-REx-TRPM3 cells, HEK293 cells expressing a tetracycline-inducible TRPM3 expression cassette, with pregnenolone sulfate increased AP-1 activity by 15.7-fold, whereas in the presence of the PIP5K inhibitor ISA-2011B, only a 2.7-fold increase in AP-1 activity was observed. Thus, the administration of ISA-2011B resulted in an 88% inhibition of TRPM3 signaling, as measured by the Coll.luc sensor.

### 2.4. Pharmacological Inhibition of Phosphatidylinositol 4,5-Bisphosphate Biosynthesis Interferes with Signaling via the Voltage-Gated Ca_v_1.2. Ca^2+^ Channel

The activity of voltage-gated Ca^2+^ channels depends on the presence of phosphatidylinositol 4,5-bisphosphate in the plasma membrane, as shown by experiments resulting in the dephosphorylation of phosphatidylinositol 4,5-bisphosphate by an inositol lipid 5′-phosphatase [40], which converts phosphatidylinositol 4,5-bisphosphate into phosphatidylinositol 4-phosphate. Figure 3c shows the modular structure of Ca_v_1.2 L-type voltage-gated Ca^2+^ channels, which consist of five subunits, the pore-forming α1 subunit, and three auxiliary subunits α2δ, β, and γ. The figure clearly shows that the modular structure of Ca_v_1.2 channels is completely different from that of the TRP channels, TRPM8 and TRPM3. Nevertheless, Ca_v_1.2 and the TRPM8 and TRPM3 channels require phosphatidylinositol 4,5-bisphosphate for activation. As a further control for the previous experiments, we analyzed whether the inhibition of PIP5Kα impaired Ca_v_1.2 Ca^2+^ channel signaling. As a measure of Ca_v_1.2 Ca^2+^ channel activity, we determined the activation of AP-1 in Ca_v_1.2 channel-expressing insulinoma cells [13,32]. Figure 3d shows that the administration of KCl and the compound FPL64176 to INS-1 832/13 insulinoma cells resulted in the strong activation of Ca_v_1.2 Ca^2+^ channels, which increased the AP-1 transcription factor activity by 6.9-fold. Inhibition of phosphatidylinositol 4,5-bisphosphate biosynthesis with the PIP5Kα inhibitor ISA-2011B significantly reduced signaling via the Ca_v_1.2-Ca^2+^ channel in the insulinoma cells. Only a 1.9-fold increase in AP-1 activity was measured. The administration of ISA-2011B resulted in an 84% inhibition of Ca_v_1.2-Ca2+ channel signaling. We conclude that inhibition of PIP5K strongly interferes with the signaling of TRPM8, TRPM3, and the Ca_v_1.2 channel.

### 2.5. Overexpression of Regulator of G-Protein Signaling-2 (RGS2) Blocks the Activation of AP-1 after Stimulation of TRPM8 Channels

**Figure 4:**
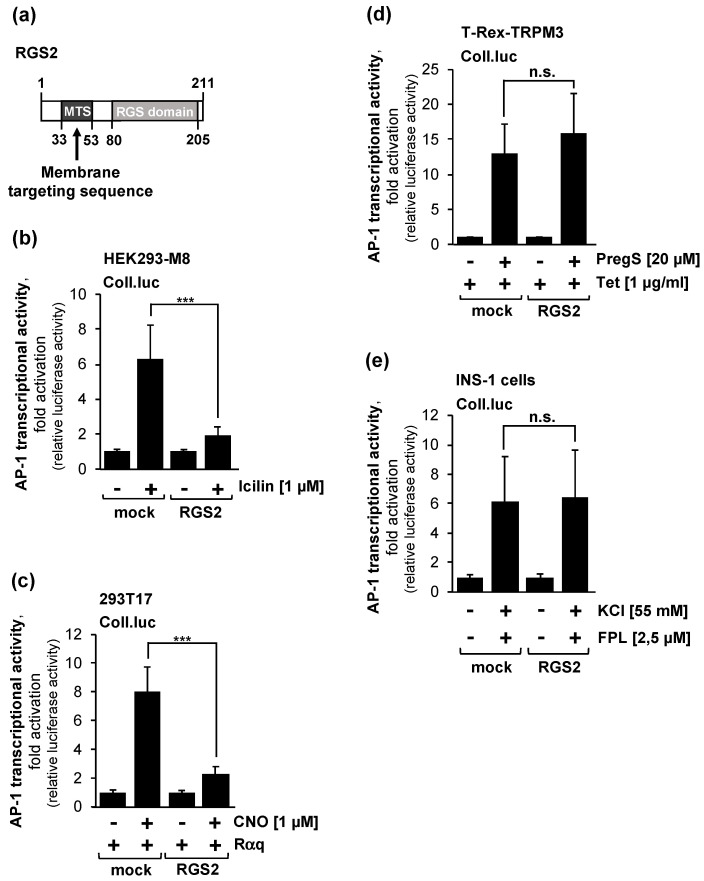
RGS2 expression reduces signal transduction after stimulation of TRPM8 channels or Gαq-coupled designer receptors. (**a**) Modular structure of RGS2. The RGS domain is responsible for binding to Gαq, while the N-terminal domain is required for targeting the protein to the plasma membrane. (**b**) HEK293-M8 cells containing the Coll.luc reporter gene were infected with a recombinant lentivirus encoding either RGS2 or β-galactosidase (mock). Cells were serum-starved for 24 h, and then stimulated with icilin (1 μM) in serum-reduced medium for 24 h. Cells were harvested and analyzed as described in the legend to Figure 2 (n = 3, *** *p* < 0.001). (**c**) HEK293 cells were infected with a lentivirus encoding the Gαq-coupled designer receptor Rαq. Cells were additionally infected with a lentivirus containing the Coll.luc reporter gene. Furthermore, the cells were infected with a lentivirus encoding either RGS2 or β-galactosidase (mock). We incubated the cells in medium containing 0.05% serum for 24 h. Stimulation of the cells was performed with CNO (1 μM) for 24 h in serum-reduced medium. Cells were harvested and analyzed as described in the legend to Figure 2 (n = 5, *** *p* < 0.001). (**d**) T-REx-TRPM3 cells containing a chromatin-integrated Coll.luc reporter gene were serum-starved for 24 h in the presence of tetracycline (1 μg/mL). The serum-starved cells were infected with a lentivirus encoding either RGS2 or β-galactosidase (mock). Cells were stimulated with pregnenolone sulfate (20 μM) for 24 h. Cells were harvested and analyzed as described in the legend to Figure 2 (n = 3; n.s., not significant). (**e**) INS-1 832/13 insulinoma cells were infected with a lentivirus containing the reporter gene Coll.luc. Additionally, we infected the cells with a lentivirus encoding either RGS2 or β-galactosidase (mock). Cells were incubated in medium containing 0.5% serum and 2 mM glucose for 24 h, and then stimulated with KCl (25 mM) and the FPL64176 (2.5 μM) for 24 h. Cells were harvested and analyzed as described in the legend to Figure 2 (n = 3; n.s., not significant).

It has been proposed that the activated GTP-bound Gαq subunit of trimeric Gq proteins binds directly to TRPM8 channels, resulting in an inhibition of channel activity [22]. To test this assumption, we expressed the regulator of G protein signaling-2 (RGS2) in HEK293-M8 cells, stimulated the cells with icilin, and measured the AP-1 activity using the Coll.luc sensor. RGS2 (Figure 4a) accelerates the rate of hydrolysis of GTP-Gαq to GDP-Gαq. The inactive GDP-Gαq then binds to Gβγ and forms an inactive trimeric G protein [41]. Figure 4b shows that the expression of RGS2 in the HEK293-M8 cells impaired the icilin-induced activation of AP-1. The stimulation of the cells with icilin increased the AP-1 activity by 6.3-fold. In the presence of RGS2, only a 2-fold increase in AP-1 activity was measured. Thus, the expression of RGS2 decreased the AP-1 activity by 82% in icilin-stimulated HEK293-M8 cells. These results suggest that the stimulation of TRPM8 channels with icilin requires an activated Gαq subunit. As a positive control, we show that the overexpression of RGS2 reduced the signaling of Gαq-coupled receptors in the order of 82% (Figure 4c). The stimulation of the cells with CNO induced AP-1 activity 8-fold, while stimulation of only 2,3-fold was measured in the presence of RGS2. The overexpression of RGS2 did not alter the signaling pathways following the stimulation of either TRPM3 channels (Figure 4d) or voltage-gated Ca_v_1.2 Ca^2+^ channels (Figure 4e).

### 2.6. Pharmacological Inhibition of Gβγ Interferes with Signaling via the TRPM3 and TRPM8 Channels

**Figure 5:**
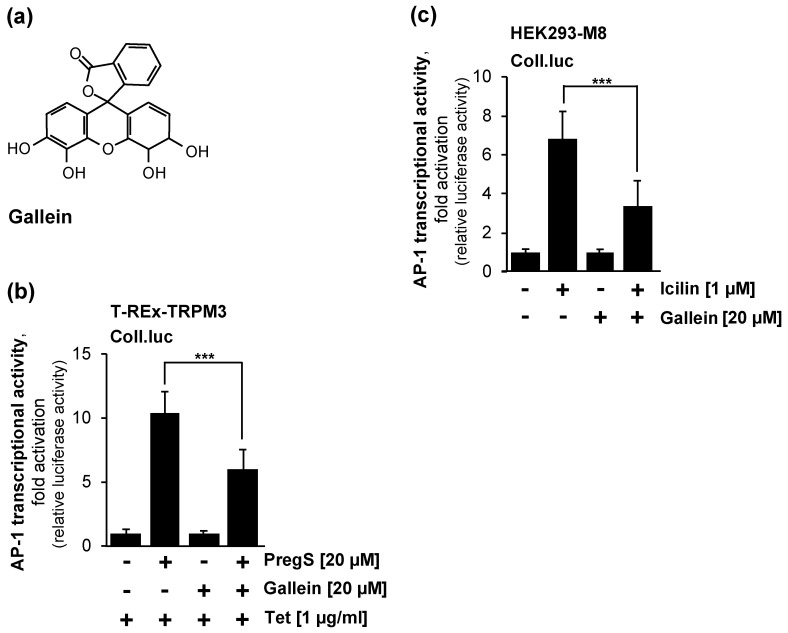
The Gβγ inhibitor gallein attenuates signal transduction mediated by TRPM8 and TRPM3 channels. (**a**) Chemical structure of gallein. (**b**) T-REx-TRPM3 cells containing a chromatin-embedded Coll.luc reporter gene were serum-starved for 24 h in the presence of tetracycline (1 μg/mL) to induced TRPM3 expression. Serum-starved cells were preincubated with gallein (20 μM) for three hours, and then stimulated with pregnenolone sulfate (20 μM) for 24 h in the presence of the compound. Cells were harvested and analyzed as described in the legend to Figure 2 (n = 3; *** *p* < 0.001). (**c**) HEK293-M8 cells containing the Coll.luc reporter gene integrated into the chromatin were serum-starved for 24 h, preincubated for 3 h with gallein (20 μM), and then stimulated with icilin (1 μM) in serum-reduced medium in the presence of gallein. Cells were harvested and analyzed as described in the legend to Figure 2 (n = 3; *** *p* < 0.001).

TRPM3 ion channels are regulated by the Gβγ subunits of trimeric Gq proteins [39,42,43]. Other Ca^2+^ channels also rely on Gβγ for activation [44,45]. We used a pharmacological approach (Figure 5a), as suggested in [43], to confirm this observation. Figure 5b shows that the stimulation of T-REx-TRPM3 cells with pregnenolone sulfate increased the AP-1 activity by 10.4-fold, while a 5.2-fold increase in AP-1 activity was observed in the presence of the Gβγ inhibitor gallein. Thus, the administration of gallein led to a 46% inhibition of TRPM3 signaling. For TRPM8, Gαq but not Gβγ was suggested to induce an inhibition in excised patches [22]. We examined the effect of gallein on icilin-induced TRPM8 signaling. Figure 5c shows that the stimulation of HEK293-M8 cells with icilin increased the AP-1 activity by 6.9-fold. In the presence of gallein, the AP-1 activity increased 3.3-fold. Thus, icilin-induced AP-1 activity was reduced by 60% in the HEK293-M8 cells in the presence of gallein.

### 2.7. The Transcription Factor c-Jun or c-Jun-Dimeriziation Proteins Is Essential for the Activation of AP-1 after Stimulation of TRPM8 Channels with Icilin

After the stimulation of TRPM8 cells with icilin, Ca^2+^ ions enter the cells through the channel. The extracellular signal-regulated protein kinase ERK1/2 acts as a signal transducer to extend the signaling cascade through the cytoplasm into the nucleus, where gene regulatory proteins are regulated via phosphorylation [12,13]. Recently, we showed that the stimulation of HEK293-M8 cells with icilin increased the expression of c-Fos [12], one of the bZIP transcription factors that form the AP-1 transcription factor complex. AP-1 was originally described as a dimer of the bZIP proteins c-Fos and c-Jun [46].

**Figure 6:**
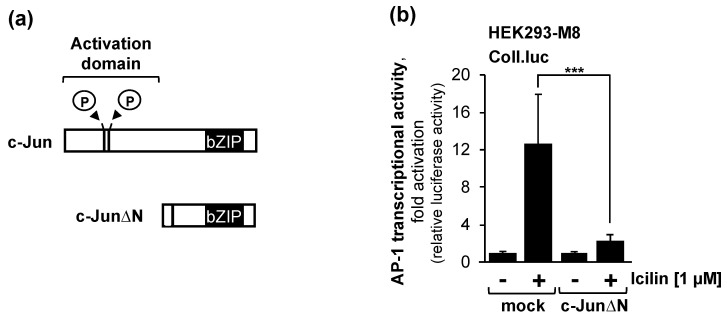
The transcription factor c-Jun is required to link the stimulation of TRPM8 with the activation of AP-1. (**a**) Modular structure of the transcription factor c-Jun and its dominant-negative c-Jun mutant c-JunΔN. c-JunΔN contains the C-terminal amino acids 188 to 331 of c-Jun, which comprise the bZIP domain. The mutant lacks the transcriptional activation domain. (**b**) Expression of c-JunΔN attenuates icilin-induced activation of AP-1 in HEK293-M8 cells. Cells were infected with a lentivirus containing a Coll.luc reporter gene. Cells were additionally infected with a lentivirus encoding either c-JunΔN or β-galactosidase (mock). Serum-starved cells were stimulated with icilin (1 μM) for 24 h. Cells were harvested and analyzed as described in the legend to Figure 2 (n = 3; *** *p* < 0.001).

Therefore, we investigated whether the c-Fos dimerization partner c-Jun is also involved within the TRPM8-induced signaling cascade to generate an active AP-1 complex. We expressed a dominant-negative mutant of c-Jun termed c-JunΔN in HEK293-M8 cells. The c-Jun mutant had no transcriptional activation domain and was, therefore, unable to activate gene transcription. c-JunΔN retained the bZIP domain, which is responsible for DNA binding and dimerization (Figure 6a). The biological activity of c-JunΔN has been demonstrated in various control experiments [24,29]. Figure 6b shows that the expression of c-JunΔN significantly reduced the AP-1 activity in the HEK293-M8 cells after the stimulation of the cells with icilin. Stimulation of HEK293-M8 cells with icilin increased the AP-1 activity by 12.6-fold. In the presence of c-JunΔN, the AP-1 activity increased only 2.3-fold. Thus, the icilin-induced AP-1 activity was reduced by 89% in the HEK293-M8 cells in the presence of c-JunΔN. We conclude that c-Jun or a c-Jun dimerization partner is an essential signaling molecule required for the activation of AP-1 within the TRPM8-induced signaling cascade. 

## 3. Discussion

In this study, we analyzed the TRPM8-induced signaling pathway that induces a change in gene transcription. Several signaling molecules have been identified in recent years [12,13,14,15], but the description of TRPM8 signaling still contains several gaps and open questions. We focused on the roles of phosphatidylinositol 4,5-bisphosphate and trimeric G protein subunits in the regulation of TRPM8 signaling. Finally, we analyzed the impact of c-Jun, a bZIP transcription factor, on the TRPM8-induced activation of AP-1.

It has been proposed that most TRP channels, along with other ion channels, are regulated by the lipid signaling molecule phosphatidylinositol 4,5-bisphosphate [47]. A variety of experimental strategies have been used to test this concept, including pharmacological and genetic methods. “Science progresses by convergence of evidence from independent observations” [47], and converging pharmacological and genetic data usually provide a clear picture. The most convincing results concerning the regulation of TRPM8 by phosphatidylinositol 4,5-bisphosphate were obtained with the use of sophisticated electrogenetic and chemical genetic tools to reduce plasma membrane phosphatidylinositol 4,5-bisphosphate levels by dephosphorylation, including the use of the rapamycin-inducible 4,5-phosphoinositide phosphatase pseudojanin and the voltage-activatable phosphatase ci-VSP. These experiments conclusively demonstrated that the activation of TRPM8 and TRPM3 requires phosphatidylinositol 4,5-bisphosphate [19,20,21,39,48]. Similar results were shown for the voltage-gated Ca^2+^ channel Ca_v_1.2 [40]. In contrast, pharmacological tools might produce questionable results due to their nonspecific activities. The application of lipids to patch membranes can lead to non-specific physicochemical changes. The addition of MgATP to excised inside-out patches has been used to activate phosphatidylinositol 4-kinase [39,48], but may also lead to the activation of other kinases. The compound wortmannin, known as an inhibitor of phosphatidylinositol-3-kinase and myosin light-chain kinase, has been used as a phosphatidylinositol 4-kinase inhibitor to decrease the concentration of phosphatidylinositol 4,5-bisphosphate [3,16,21,39,48]. In our study, the application of wortmannin did not inhibit TRPM3 signaling at all. Rather, we observed an increase in AP-1 activity after the stimulation of the TRPM3 channels with pregnenolone sulfate in the presence of 35 μM wortmannin (G.Thiel, unpublished observations). In this study, we used the compound ISA-2011B for inhibiting PIP5K [27,28], the main phosphatidylinositol 4,5-bisphosphate-synthesizing enzyme. The results showed that the administration of this compound strongly reduced the signaling mediated by the TRPM8 and TRPM3 channels. The administration of ISA-2011B to T611 cells expressing TRPC6 channels also significantly inhibited the hyperforin-induced activation of AP-1 (G.Thiel, unpublished observations), supporting the view that the PIP5K-catalyzed biosynthesis of phosphatidylinositol 4,5-bisphosphate is essential for the activation of numerous TRP channels. Finally, in this study, we demonstrated that the administration of ISA-2011B strongly reduced signaling through the voltage-gated Ca_v_1.2 Ca^2+^ channel, confirming the previous suggestion that phosphatidylinositol 4,5-bisphosphate is a cofactor required for full Ca_v_1.2 channel activity [40]. These results put the spotlight on PIP5K as an important regulator of TRP channel signaling via the regulation of phosphatidylinositol 4,5-bisphosphate biosynthesis. It would be interesting to know if voltage-clamp and Ca^2+^ imaging techniques could confirm that ISA-2011B inhibits TRPM8, TRPM3, and Ca_v_1.2 Ca^2+^ channel activites.

Phosphatidylinositol 4,5-bisphosphate is thought to interact with ion channels via electrostatic interactions or by direct binding to specific binding sites within the channel proteins. Recently published structural data provide a detailed view of the binding of phosphatidylinositol 4,5-bisphosphate to the TRPM8 and TRPM3 channels. Structural data suggest a phosphatidylinositol 4,5-bisphosphate binding site involving the TRP domain, the pre-S1 domain, and the melastatin homology region-4 (MHR4) of the adjacent subunit [26]. A similar binding site has been proposed for the TRPM3 channel, involving amino acid residues within the pre-S1 segment, the S4-S5 linker, and the TRP domain [48,49].

The stimulation of Gαq-coupled receptors has been suggested to impair the activation of TRPM8 and TRPM3 [17,21] via the activation of PLCβ, leading to the hydrolysis of phosphatidylinositol 4,5-bisphosphate. Similarly, the stimulation of TrkA or PDGBβ receptors, which stimulates PLCγ, has been shown to inhibit TRPM8 current [16,17]. In these studies, indirect evidence for a direct relationship between receptor stimulation and phosphatidylinositol 4,5-bisphosphate hydrolysis was provided by using the translocation of the PLCγ-PH domain from the plasma membrane to the cytoplasm as a biosensor. This translocation assay, i.e., the loss of membrane localization of the biosensor, should be treated with caution [50]. The PLCγ-PH domain does not specifically bind to phosphatidylinositol 4,5-bisphosphate, but interacts 20-fold more strongly with IP_3_ [50,51]. Thus, the PLCγ-PH domain could act as an IP_3_ sponge and attenuate IP_3_-mediated downstream signaling. Much stonger binding to phosphatidylinositol 3,4,5-trisphosphate than that to phosphatidylinositol 4,5-bisphosphate has also been reported [52]. Another study showed that increased intracellular Ca^2+^ concentrations, as occurring after the stimulation of Gαq-coupled receptors or TRP channels, interfere with PLCγ-PH binding to the membrane [53]. Decorating the plasma membrane with a PLCγ-PH domain protein may sequester its targets and interfere with the binding of other phosphatidylinositol 4,5-bisphosphate-binding proteins. As a result, intracellular signaling pathways downstream of TRP channels would be disrupted and off-target effects may occur. For example, the expression of the PH domain of PLCβ inhibits the activation of PLCβ by Gβγ [54], the expression of the PH domain of PLCγ1 inhibits the stimulation of PLC by platelet-derived growth factor [52], and the expression of the PH domain of PLCδ_1_ reduces the concentration of PIP5K in the plasma membrane [55].

In contrast to the hypothesis that the activation of Gαq-coupled receptors inhibit TRPM8 activation via a transient reduction in phosphatidylinositol 4,5-bisphosphate levels, the direct binding of the inactive and activated Gαq subunit to TRPM8 channels has been proposed. Activated Gαq forms a complex with TRPM8 channels and, in this way, directly inhibits the activation of TRPM8 after the stimulation of Gαq-coupled receptors, independent of the downstream PLC pathway [22]. Overexpression experiments showed that a Gαq mutant that lacked intrinsic GTPase activity and was, therefore, in its active, GTP-bound conformation very actively inhibited TRPM8 current. The direct binding of TRPM8 and Gαq has also been reported by others [23]. Furthermore, this study demonstrated that the stimulation of TRPM8 leads to dissociation, i.e., the activation of trimeric G-proteins and the subsequent activation of PLC.

In this study, we used a genetic approach to inhibit the activity of Gαq. We expressed the regulator of G-protein signaling-2 (RGS2) in the cells, which stimulates the GTPase activity of Gαq and, thus, inactivates Gαq, which, in its GDP-bound state, forms a complex with the Gβγ subunits. The expression of RGS2 strongly inhibited the signaling of a Gαq-coupled designer receptor, clearly demonstrating its activity. Similarly, the expression of RGS2 strongly inhibited TRPM8 signaling, suggesting that the stimulation of TRPM8 requires an activated Gαq subunit. Thus, it is not the activated Gαq that inhibits TRPM8, as suggested [22], but rather the inactivation of Gαq. The overexpression of RGS2 had no effect on TRPM3 or Ca_v_1.2 signaling, indicating clear differences in the regulation of TRPM8 and TRPM3/Ca_v_1.2 signaling. Similarly, the overexpression of RGS2 was shown to reduce the Ca^2+^ and Na^+^ current of TRPV6, whereas the activity of the TRPV5 channel was unaffected by RGS2 [56]. Thus, a subset of TRP channels is regulated by an activated Gαq subunit and, therefore, responds to the overexpression of RGS2 (TRPM8 and TRPV6), while others are completely inert with RGS2 overexpression and do not require Gαq (TRPM3 and TRPV5).

Recently, the TRPM3 ion channel has been shown to be regulated by the Gβγ subunits of trimeric Gq proteins [39,42,43], and a binding site has been proposed [57]. In this study, we used a pharmacological approach to confirm that the Gβγ subunits modulate the activity of TRPM3 channels. Furthermore, we demonstrated that TRPM8 channels also respond to the Gβγ-inhibitor gallein. However, the proposed Gβγ binding site for TRPM3 is absent in TRPM8, suggesting that the regulatory role of Gβγ for TRPM8 is mediated by other interactions. It is possible that the Gβγ subunits do not interact directly with TRPM8, but regulate the activity of TRPM8 by manipulating the activity of PLCβ. We conclude that TRPM8 channel activity depends on both an activated Gαq subunit and Gβγ subunits.

What role does PLC play in regulating TRPM8 activity and signaling? It has been proposed that many TRP channels “are either activated downstream of the PLC pathway, or modulated by it” [58]. An influx of Ca^2+^ ions through the TRPM8 channel has been shown to activate PLCδ, which regulates TRPM8 activity by inducing a depletion of the phosphatidylinositol 4,5-bisphosphate levels in the plasma membrane [16]. In contrast, it has been suggested that an activated Gαq-subunit inhibits TRPM8 independently of PLC [22]. The fact that the activation of TRPM8 requires Gβγ and an activated Gαq suggests that PLCβ is subsequently activated, leading to a reduction in the phosphatidylinositol 4,5-bisphosphate concentration. The Gq subunits can simultaneously and independently bind to PLCβ and modulate its activity. Gαq changes the autoinhibition mediated by the X-Y linker of PLCβ, leading to an increased *k_cat_* of PLCβ. Gαq could also support the orientation of the catalytic core of the enzyme at the membrane [59]. The Gβγ subunits activate PLCβ by recruiting it to the membrane, i.e., bringing it closer to its lipid substrate [60]. However, due to experimental problems and the use of indirect assay systems, it is difficult to quantify the reduction in the phosphatidylinositol 4,5-bisphosphate concentration after PLC activation and to determine the time frame until the original phosphatidylinositol 4,5-bisphosphate concentration is restored by resynthesis from phosphatidylinositol 4-phosphate. The stimulation of Gαq-coupled receptors has been shown to cause only a small, transient decrease in the total amount of phosphatidylinositol 4,5-bisphosphate, which is efficiently replenished by phosphatidylinositol 4,5-bisphosphate-synthesizing enzymes [61,62]. Recently, we showed that the C-terminal domain of PLCβ1 and PLCβ3 interacts with plasma membrane targets, most likely phosphatidylinositol 4,5-bisphosphate, and blocks the biological activation of TRPM8 channels [15]. It has been suggested that about two-thirds of the phosphatidylinositol 4,5-bisphosphate pool is sequestered by binding proteins and is not freely available for phosphatidylinositol 4,5-bisphosphate effector proteins [63]. Therefore, PLCβ enzymes might regulate TRPM8 activation by masking phosphatidylinositol 4,5-bisphosphate with its C-terminal domain. It is tempting to speculate that the binding of Gβγ and Gαq to PLCβ enzymes induces a conformational switch of PLCβ that removes this blockade and allows for the activation of TRPM8 channels.

The pathway from the plasma membrane to the nucleus ends with the activation of stimulus-responsive transcription factors. In this study, we were able to show that the transcription factor c-Jun is essential for the formation of an active AP-1 complex within TRPM8-induced signaling. Recently, we showed that expression of the c-Jun dimerization parter c-Fos is upregulated upon the stimulation of TRPM8 [12]. AP-1 controls numerous biological activities, including the regulation of proliferation, differentiation, and cell death [64]. The outcome often depends on the cell type. TRPM8 is associated with tumor development, for example, prostate cancer, colon cancer, and squamous cell carcinoma [8,9]. In this context, it is of particular interest to investigate the oncogenic role of c-Jun within the TRPM8-induced signaling cascade. This also provides an indication of where future studies on c-Jun-regulated genes could be directed.

## 4. Materials and Methods

### 4.1. Cell Culture and Reagents

HEK293 cells expressing either TRPM3 (T-REx-TRPM3 cells) or TRPM8 (HEK293-M8 cells) have been described elsewhere [65,66]. HEK293T/17 cells were infected with a lentivirus to express Rαq, a Gαq-coupled designer receptor, as described [33]. HEK293-M8 cells, T-REx-TRPM3 cells, and HEK293 cells expressing Rαq were incubated in DMEM containing 0.05% fetal bovine serum for 24 h prior to stimulation. Stimulation was performed with icilin (PubChem CID: 161930; 1 μM, Santa Cruz Biotechnology, Heidelberg, Germany, # sc-201557, dissolved in DMSO), pregnenolone sulfate (PubChem CID: 105074; PregS, 20 μM, dissolved in DMSO, Sigma-Aldrich GmbH, Taufkirchen, Germany, # P162), or clozapine-*N*-oxide (PubChem CID: 135445691; 1 μM CNO, dissolved in ethanol, Enzo Life Sciences, Lörrach, Germany, # NS-105-0005), respectively, for 24 h in medium containing 0.05% fetal bovine serum. INS-1 832/13 insulinoma cells were a kind gift from Hindrik Mulder [67], Lund University, Sweden, with the permission of Hans-Ewald Hohmeier and Christopher Newgard, Duke University, USA. The cells were cultured in RPMI 1640 medium containing 10% fetal calf serum, 2 mM glucose, 10 mM HEPES, 2 mM L-glutamine, 1 mM sodium pyruvate, 50 µM β-mercaptoethanol, 100 units/mL penicillin, and 100 µg/mL streptomycin. The cells were incubated for 24 h in DME medium containing 0.5% fetal calf serum, 2 mM glucose, 10 mM HEPES, 2 mM L-glutamine, 1 mM sodium pyruvate, 50 µM β-mercaptoethanol, 100 units/mL penicillin, and 100 µg/mL streptomycin before stimulation. The stimulation of INS-1 832/13 cells with KCl (PubChem CID: 4873; 55 mM) and FPL64176 (PubChem CID: 3423; 2.5 μM, dissolved in DMSO, a kind gift from Alomone Labs, Israel, Cat #: F-160) was performed in the same medium for 24 h in the presence of the inhibitory compound. The cells were preincubated for 3 h with the PIP5α inhibitor ISA-2011B, a diketopiperazine fused C-1 indol-3-yl substituted tetrahydroisoquinoline (PubChem CID: 49853637; MedChemExpress, Monmouth, NJ, USA, Cat. No. HY-16937, dissolved in DMSO) at a concentration of 10 μM, or gallein (PubChem CID: 73685, Santa Cruz Biotechnology, Inc., Heidelberg, Germany Ct.-No. sc-202631, dissolved in DMSO) at a concentration of 20 μM. The cells were stimulated for 24 h in the presence of these compounds.

### 4.2. Lentiviral Gene Transfer

The lentiviral transfer vectors pFUW-HA-Rαq, which encodes the Gαq-coupled designer receptor Rαq, pFUW-HA-RGS2, encoding HA-tagged RGS2, and pFUW-c-JunΔN, encoding a dominant-negative mutant of c-Jun, respectively, have been described elsewhere [24,29,33]. The lentiviral transfer vector pFWColl.luc has been described [34]. Viral particles were produced by the triple transfection of HEK293-TN cells with the gag-pol-rev packaging plasmid, the pCMVG plasmid encoding the glycoprotein of vesicular stomatitis virus, and a lentiviral transfer vector.

### 4.3. Reporter Gene Assay

The infected cells were maintained in medium containing 0.05% fetal bovine serum for 24 h prior to stimulation for 24 h. The cell extracts were prepared using reporter lysis buffer (Promega, Mannheim, Germany) and assayed for luciferase activities that were normalized to the protein concentrations of the extracts. The luciferase activities were measured using a luminometer (Berthold Detection Systems, Pforzheim, Germany). The protein concentrations of the extracts were determined using a BCA protein assay kit (Thermo Fisher Sci., Waltham, MA, USA).

### 4.4. Statistics

The data are shown as the means +/− SD of at least three independent experiments performed in quadruplicate. The two-tailed Student’s *t*-test was used for the statistical analyses. Statistical probability is expressed as *** *p* < 0.001, ** *p* < 0.01, and * *p* < 0.05. We considered values significant when *p* < 0.05.

## 5. Conclusions

**Figure 7:**
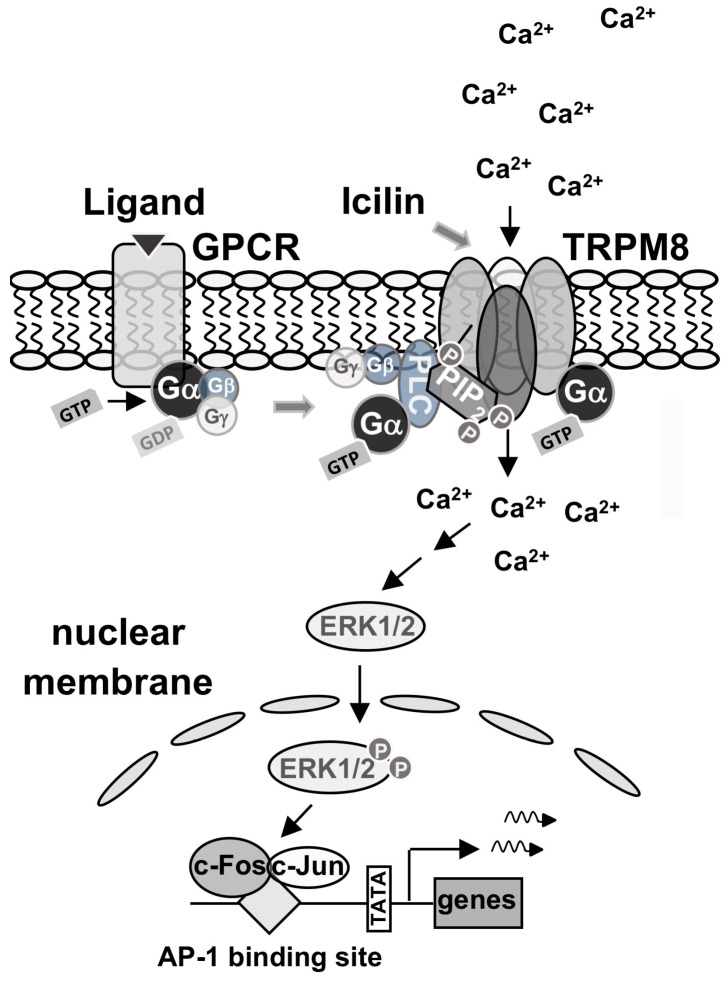
Signal transduction of TRPM8 channels. The TRPM8 channel is a tetramer embedded into the plasma membrane. The channel interacts with phosphatidylinositol 4,5-bisphosphate, which is essential for its activation. Reducing phosphatidylinositol 4,5-bisphosphate levels by blocking its biosynthesis with a PIP5K inhibitor impairs TRPM8 activation. PIP5K is, therefore, an important regulator of TRPM8 signaling. The stimulation of Gαq-coupled receptors triggers the dissociation of the trimeric G proteins into a GTP-bound α-subunit and the βγ subunits. The Gα subunit interacts with TRPM8 channels and is essential for TRPM8 activation. The α-subunit and the βγ subunits bind to PLCβ and activate the enzyme by increasing *k_cat_* and changing the orientation of the enzyme in the membrane to its substrate. This conformational change may remove the blockade of PLCβ to TRPM8 channels. After the activation of TRPM8, Ca^2+^ ions flow into the cells through the channel and trigger the activation of ERK1/2, which acts as signal transducer. The kinase translocates into the cell nucleus and activates AP-1, which is composed of the bZIP proteins c-Jun and c-Fos.

The stimulation of TRPM8 channels induces an intracellular signaling pathway that leads to the activation of stimulus-responsive transcription factors that alter the gene expression pattern of cells. In this study, we analyzed the signaling molecules required for the link between the stimulation of TRPM8 channels and gene transcription, and a summary of the results is depicted in Figure 7. We focused on the roles of phosphatidylinositol 4,5-bisphosphate and trimeric G-protein subunits within the TRPM8-induced signaling cascade. The TRPM8 channel interacts with the lipid mediator phosphatidylinositol 4,5-bisphosphate, which is essential for its activation. Reducing phosphatidylinositol 4,5-bisphosphate levels by blocking its biosynthesis with a PIP5K inhibitor impaired TRPM8 activation. These data put PIP5K into the limelight as an important regulator of TRPM8 signaling. It has been suggested that the stimulation of G protein-coupled receptors can modulate TRPM8 activation, although this issue is controversial. The stimulation of Gαq-coupled receptors triggers the dissociation of the trimeric G proteins into a GTP-bound α-subunit and the βγ subunits. The Gα subunit has been shown to interact with TRPM8 channels. This study showed that the activated GTP-bound form of Gαq is essential for TRPM8 signaling. Similarly, we showed that the inhibition of the βγ subunits also impaired TRPM8 signaling. Future studies should show whether Gβγ interacts directly with TRPM8 or carries out its activity via influencing PLCβ. Both the α-subunit and the βγ subunits bind simultaneously to PLCβ and activate the enzyme by recruiting it to the membrane, increasing *k_cat_* and changing the orientation of the enzyme to its substrate. The C-terminal domains of PLCβ1 and PLCβ3 block the biological activation of TRPM8 channels. We propose that the binding of Gβγ and Gαq to PLCβ enzymes causes a conformational change in the enzyme that removes this blockade and enables the activation of TRPM8 channels. After the stimulation of TRPM8, Ca^2+^ ions flow through the channel into the cytoplasm and trigger the activation of the signal transducer ERK1/2. The phosphorylated and activated form of ERK1/2 migrates into the cell nucleus and activates AP-1, which consists of the bZIP proteins c-Jun and c-Fos.

## Data Availability

Data are contained within the article.
